# Dna2 processes behind the fork long ssDNA flaps generated by Pif1 and replication-dependent strand displacement

**DOI:** 10.1038/s41467-018-07378-5

**Published:** 2018-11-16

**Authors:** Silvia Emma Rossi, Marco Foiani, Michele Giannattasio

**Affiliations:** 1 IFOM (Fondazione Istituto FIRC di Oncologia Molecolare), Via Adamello 16, Milan, 20139 Italy; 2 Dipartimento di Oncologia ed Emato-Oncologia, Universita’ degli Studi di Milano, Via Festa del Perdono 7, Milan, 20122 Italy

## Abstract

Dna2 is a DNA helicase-endonuclease mediating DSB resection and Okazaki fragment processing. Dna2 ablation is lethal and rescued by inactivation of Pif1, a helicase assisting Okazaki fragment maturation, Pol32, a DNA polymerase δ subunit, and Rad9, a DNA damage response (DDR) factor. Dna2 counteracts fork reversal and promotes fork restart. Here we show that Dna2 depletion generates lethal DNA structures activating the DDR. While *PIF1* deletion rescues the lethality of Dna2 depletion, *RAD9* ablation relieves the first cell cycle arrest causing genotoxicity after few cell divisions. Slow fork speed attenuates DDR in Dna2 deprived cells. Electron microscopy shows that Dna2-ablated cells accumulate long ssDNA flaps behind the forks through Pif1 and fork speed. We suggest that Dna2 offsets the strand displacement activity mediated by the lagging strand polymerase and Pif1, processing long ssDNA flaps to prevent DDR activation. We propose that this Dna2 function has been hijacked by Break Induced Replication in DSB processing.

## Abstract (web summary)

DNA2 encodes a 5′ flap DNA endonuclease involved in replication and DNA double strand break processing. Here the authors by using a conditional degron system together with electron microscopy characterize the role played by Dna2 and Pif1 helicase during unperturbed DNA replication in *S. cerevisiae*.

## Introduction

Okazaki fragment maturation depends on the strand displacement activity of Pol δ, which peels off the 5’end of the previous Okazaki fragment, Rad27/FEN1, which cuts the resulting DNA flap, and DNA nick ligation mediated by the DNA ligase I^1–4^. While RnaseH appears to have an important role in RNA–DNA primer removal during Okazaki fragment processing in *Escherichia coli*^5^, *Saccharomyces cerevisiae* cells deleted for the gene encoding RNAase H2 (*RNH35*) are viable, suggesting that direct RNA–DNA primer removal is not essential in budding yeast^6^. Importantly, *rad27 rnh35* mutant cells do not support viability, suggesting that Fen1 and RNAase H2 have redundant roles in Okazaki fragment processing^6^. The Polδ- dependent strand displacement of the previous Okazaki fragment, which is stimulated by proliferating cell nuclear antigen (PCNA), is counterbalanced by its 3’–5’nucleolitic proof reading activity that resects the growing 3’ end of the Okazaki fragment^2,4,7^. Mutations in the proof reading activity of DNA polymerase δ are lethal in combination with *RAD27* deletion, suggesting that DNA polymerase δ-dependent degradation of the growing 3’ ends of the Okazaki fragments and Fen1 synergize in the maturation of the Okazaki fragments^8^. Dna2 is an essential DNA helicase and 5’ flap endonuclease that cuts 5’-single-stranded DNA (ssDNA) tails created during Okazaki fragment maturation and double-strand break resection^9–15^. DNA flaps were visualized by electron microscopy in *dna2*Δ germinated spores from *Schizosaccharomyces pombe*^16^. Pif1, a DNA helicase assisting replication across pausing sites^17–21^ and Okazaki fragment maturation^22–25^, contributes to the displacement of a fraction of 5’ ends of Okazaki fragments, forming long ssDNA tails that are cleaved, sequentially, by Dna2 and Fen1, through a secondary pathway of Okazaki fragment maturation known as alternative pathway of Okazaki fragment processing (APO)^3,22,23,26–29^. Pif1 activity during Okazaki fragment maturation is performed in the context of the Pif1–PCNA–Polδ complex^30^. Fen1 and Dna2 interact with PCNA^31,32^. If the long DNA flaps generated by Pif1–PCNA–Polδ are bound by the replication protein A (RPA), they are no longer efficiently cleaved by Fen1 but, instead, they can be processed by Dna2^26,27,29^. These in vitro observations led to the proposal that, at least in vitro, Dna2 and Fen1 can act sequentially to process long DNA flaps during APO^26–29^. However, while *DNA2* is an essential gene, *rad27*Δ cells are alive^33^. It is possible that, in the absence of *RAD27*, the 5’-flap endonuclease activity of Exo1 can substitute for Fen1 in *rad27*Δ cells^34^. Moreover, *rad27*Δ cells have high rates of chromosome rearrangements and replication-associated abnormalities, suggesting that Exo1 backup of Fen1 function in Okazaki fragment processing is not so efficient although it supports viability^35^. A logical implication coming from the in vitro studies is that in the absence of Dna2, the strand displacement activities of Pif1–PCNA–Polδ would create a fraction of long DNA flaps on the lagging strand that cannot be processed by Fen1. In this context, recent work provides evidence that Dna2 can cut at the junction between ssDNA and double-stranded DNA (dsDNA) at the base of a DNA flap, suggesting that Dna2 could also work as sole nuclease in the processing of Okazaki fragments^32^. The essential role of Dna2 is still under debate. In in vitro reactions simulating Okazaki fragment processing, Dna2 was shown to cut a sub-fraction of long Pif1-dependent DNA flaps generated during lagging strand DNA synthesis^22–25,36^. Intriguingly, early electron microscopy studies showed that DNA synthesis through strand displacement, catalysed by bacteriophage DNA polymerases, induces the formation of long ssDNA flaps, suggesting that extended and processive strand displacement can be achieved either by single DNA polymerases or by “two proteins modules” constituted by a DNA helicase and a DNA polymerase^37,38^ suggesting a possible similar action by the Pif1–PCNA–Polδ complex in eukaryotic cells^30^. An inside into the essential role of Dna2 came from the discovery that the cell lethality of *DNA2-*deleted cells could be rescued by inactivation of Pif1^22^, Pol32^22^, a Polδ subunit^39^, and Rad9^40^, a checkpoint factor^41^. These genetic and in vitro observations were rationalized by proposing that, during lagging strand DNA synthesis, extended Polδ- and Pif1-dependent strand displacement events generate long DNA flaps that must be cut by Dna2^22^. Dna2 has been also implicated in counteracting fork reversal^42^ and promoting fork restart^43^.

Here we used conditional alleles of *DNA2* to show that a single unperturbed S phase, following Dna2 depletion, generates toxic and lethal DNA structures activating the DNA damage response (DDR), without inducing fork pausing and chromosome fragmentation. While *PIF1* deletion rescues cell lethality owing to Dna2 depletion, *RAD9* ablation only relieves the first cell cycle block induced by the absence of Dna2, leading to Pif1-dependent ribosomal DNA (rDNA) chromosome fragmentation after few cell divisions. Slowing down replication fork speed by hydroxyurea (HU) treatment or by lowering the temperature attenuates the DDR response and the first cell cycle arrest in cells deprived of Dna2 and suppresses the lethality of *dna2 rad9* cells. By in vivo psoralen cross-linking and electron microscopy analysis^44^ we found that *DNA2*-ablated cells experiencing one round of chromosome replication accumulate replication intermediates characterized by very long ssDNA flaps (3000 nucleotides (nts)). These flaps are rarely in the proximity of the fork branching point and their accumulation and extension depend on Pif1 and fork speed. The data presented in this work suggest that the essential role of Dna2 during an unperturbed S phase is to counteract the formation of Pif1-and fork speed-dependent DNA flaps to prevent DDR hyper-activation and chromosome fragmentation.

## Results

### One round of DNA replication in the absence of Dna2 induces  Pif1-and DNA replication fork speed-dependent DDR hyper-activation and cell cycle arrest in G2

**Fig. 1:**
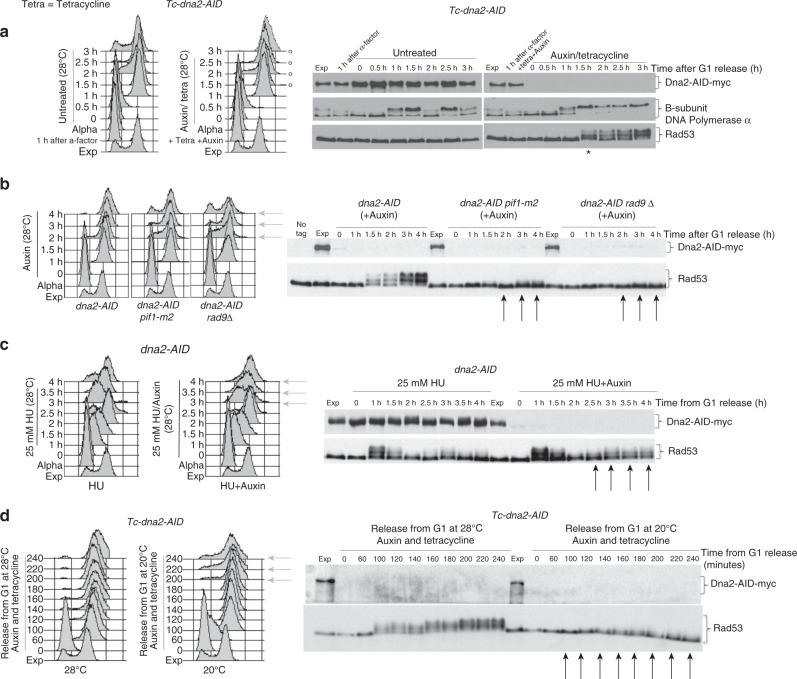
Pif1 and fork speed induce *RAD9*-dependent DDR hyper-activation in Dna2-depleted *CL-dna2* cells. **a**–**d** Dna2 was depleted in G1 in the indicated strains and cells were released into unperturbed S phase (**a**, **b**), S phase in the presence of a low dose of HU (**c**) or at a low temperature (**d**). **a**, **c** Non-depleted cells were kept in parallel as control. DNA content and the phosphorylation state of the indicated proteins were analysed, respectively,  by fluorescence-activated cell sorting (FACS) and western blotting at the indicated time points and in the specified genetic backgrounds. **a** Black asterisk and white circles indicate, respectively, Rad53 hyper-activation and first cell cycle arrest in Dna2-depleted cells. **b**–**d** Black arrows and grey arrows indicate, respectively, lack of Rad53 activation and cells that initiate the second cell cycle

We generated the conditional lethal *Tc-dna2-AID* allele (*CL-dna2*) to study the essential function of Dna2 (Fig. 1 and see Methods). *CL-dna2* mutants, experiencing one round of DNA synthesis under restrictive conditions, completed S phase with kinetics similar to control cells but arrested with a 2C DNA content, phosphorylated DNA polymerase alpha B subunit^45^, a marker of G2 arrest, and hyper-phosphorylated Rad53, indicative of DDR activation (Fig. 1a). Preventing mitosis by nocodazole addiction did not influence DDR activation, while Dna2 depletion following S-phase completion precluded Rad53 activation (Supplementary Fig. 1, A). Thus, DDR activation depends on chromosome replication but arises after S-phase completion. Late cell cycle arrest and DDR activation in Dna2-depleted cells were suppressed by *pif1* and *rad9* mutations (Fig. 1b). We then addressed whether replication fork speed influenced DDR activation in *CL-dna2* cells (Fig. 1c, d). Dna2-depleted cells experiencing a slow S phase induced by treatment with low doses of HU exhibited transient DDR activation, as in the control cells, and continued to cycle, although residual DDR activation was still detectable (Fig. 1c). When we slowed down fork speed by releasing Dna2-depleted cells into S phase at a low temperature, again DDR activation was attenuated (Fig. 1d). Hence, fast forks in the absence of Dna2 generate toxic DNA structures leading to DDR activation.

### Pif1, *RAD9*-dependent DDR and DNA replication fork speed cause cell lethality in Dna2-depleted cells

**Fig. 2:**
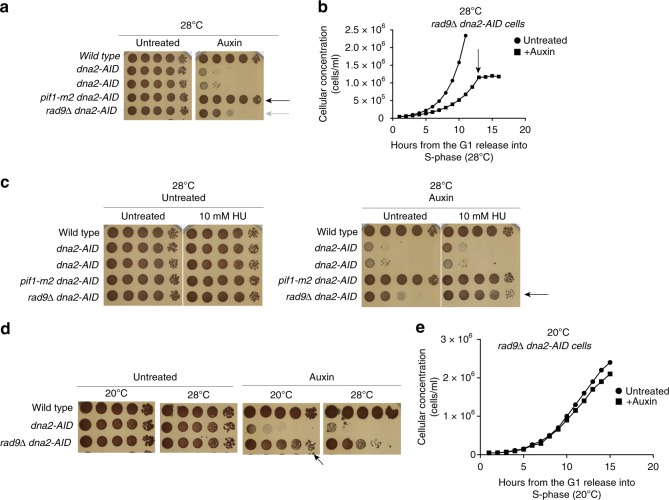
Fork speed and Pif1 induce cell death in Dna2-depleted cells. **a** In-plate cell survival assay. The indicated yeast strains were depleted (auxin) or non-depleted (untreated) for Dna2. Black and grey arrows indicate suppression of cell lethality induced by *pif1-m2* mutation or *RAD9* deletion. **b** In liquid growth curve of *dna2-AID rad9*Δ cells depleted for Dna2 in G1 (auxin) or non-depleted (untreated) and released into the cell cycle. The black arrow indicates growth arrest in *dna2-AID rad9*Δ cells depleted for Dna2. **c**, **d** In-plate cell survival assay. Black arrows indicate that low HU dose or low temperature suppress the cell lethality induced by depletion of Dna2 in *dna2-AID rad9*Δ cells. **e** Same experiment as in (**b**), but cells were released at 20 °C. In this condition, *dna2-AID rad9*Δ cells depleted for Dna2 do not arrest cell growth even after 15 h from the G1 release

While Pif1 ablation rescued the lethality of Dna2-depleted cells, *RAD9* deletion allowed *CL-dna2 rad9*Δ mutants to perform only few cell divisions on plates and liquid cultures at 28 °C (Fig. 2a, b). This is in line with previous studies showing that, although *RAD9* deletion suppresses the cell lethality of *dna2*Δ cells at low temperatures (20–23 °C), the *rad9*Δ *dna2Δ* strain is temperature sensitive and has a reduced fitness at 28–30 °C^40,46^. Overall, previous data and the observations presented in this study suggest that under fast replication conditions, Rad9, in the absence of Dna2, promotes a first cell cycle barrier preventing the propagation of genotoxicity to the next cell cycles; without Rad9, fast replication still kills Dna2-depleted cells, by causing the accumulation of lethal DNA structures after 4–5 divisions (Fig. 2b). Slowing down fork speed did not ameliorate the viability of Dna2-depleted cells, likely due to residual DDR activation (Fig. 2c, d). In fact, low HU doses or low temperatures restored cell viability in Dna2-depleted *rad9* mutants (Fig. 2c, d). We note that while slowing down replication in Dna2-depleted cells suppressed the first cell cycle arrest (Fig. 1c, d), the three major pathways causing lethality in Dna2 depleted cells (namely Pif1, DNA replication fork speed, and DNA damage checkpoint) were still functional. Hence, it is reasonable to think that a slow replication mode, in the absence of Dna2, can lead to the accumulation of Pif1-dependent toxic DNA structures that would activate the DNA damage checkpoint and generate genome damage over few cell cycles, ultimately causing cell cycle arrest or cell death. This is consistent with the finding that *RAD9* deletion ameliorates the growth of Dna2-depleted cells experiencing a slow replication (Fig. 2c, d). However, *RAD9* deletion in Dna2-depleted cells does not prevent damages caused by replication fork speed and Pif1. One possible scenario is that Pif1 creates toxic long DNA flaps in Dna2-depleted cells either by acting as an accessory strand displacement factor of Polδ (in agreement with recent studies^21,30^), or by acting, independently, in the unscheduled unwinding of Okazaki fragments through its 5’–3’ directed ssDNA translocase activity^47^.

### A single S phase in the absence of Dna2 produces long Pif1-and DNA replication fork speed-dependent DNA flaps

**Fig. 3:**
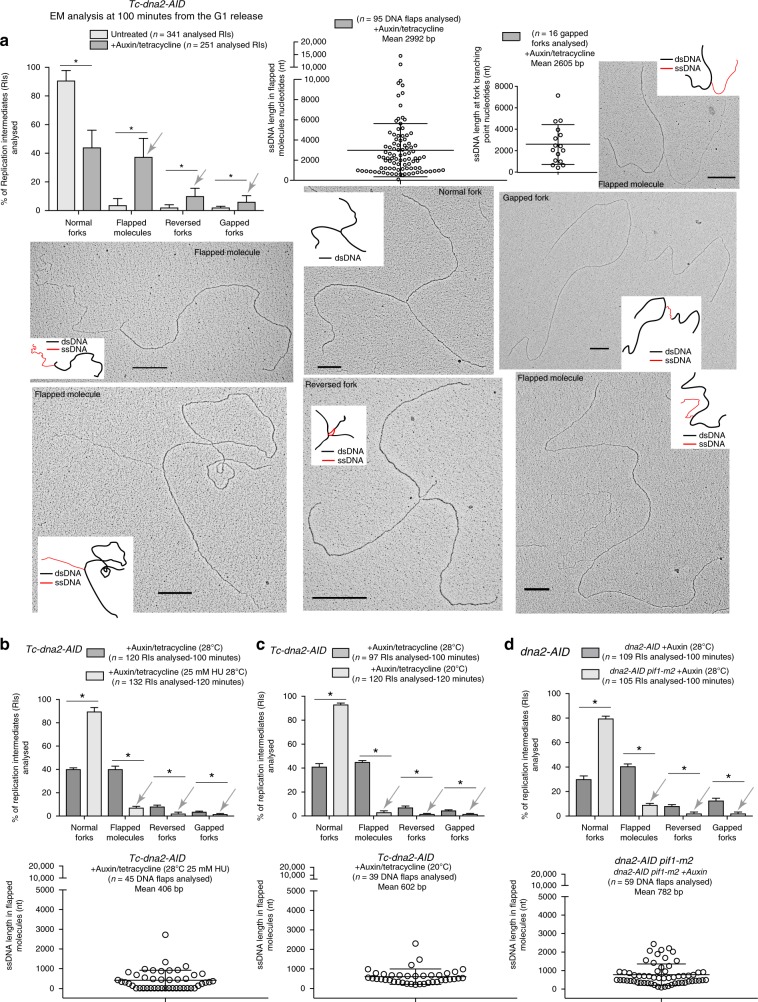
Fork speed and Pif1 induce flapped molecules, gapped forks, and reversed forks in Dna2-depleted cells. **a** The indicated *CL-dna2 (Tc-dna2-AID)* cells depleted (+auxin/tetracycline) for Dna2 in G1 or non-depleted (untreated) were released into S phase and RIs were analysed by TEM at 100 min from S-phase initiation (see also figure 1a for the conditions of this experiment). The chart reports the percentages and standard deviations of RIs found in the presence or in the absence of Dna2 (results are from three independent experiments, *n* indicates the total number of molecules analysed). *P *<* 0.05 by two-tailed *t*-test. Distributions of the lengths of the ssDNA tails in flapped molecules and ssDNA gaps in gapped forks in Dna2-depleted cells are also shown. The mean of the length of the ssDNA tails in flapped molecules or ssDNA gaps in gapped forks is reported for each chart. Grey arrows indicate abnormal RIs accumulated after a single S phase without Dna2. A representative TEM picture is shown for each type of RI found with a schematic representation of the molecule with dsDNA in black and ssDNA in red. Black scales bars of 360 nm, which correspond to 1  kilobase of DNA, are reported on each picture. **b**, **c**. As in (**a**) but percentages  of RIs and distribution of the lengths of the ssDNA tails in flapped molecules (results from two independent experiments) are reported for *tc-dna2-AID* cells after a single S phase without Dna2 (auxin/tetracycline) in the absence or presence of 25 mM of HU (**b**), or at normal (28 °C) or low temperature (20 °C) (**c**). **d** RI analysis as in (**b**, **c**) in *dna2-AID, dna2-AID pif1-m2* cells depleted for Dna2 (auxin) grown at 28 °C. **b**–**d** Grey arrows indicate suppression of accumulation of aberrant RIs in the indicated conditions and genetic backgrounds (results from two independent experiments)

We directly visualized, by in vivo psoralen cross-linking and transmission electron microscopy (TEM) analysis^44^, the replication intermediates (RIs) accumulating in Dna2-depleted cells when DDR was fully active (Fig. 1a, 3a). We analysed more than 300 RIs in *CL-dna2* cells grown under permissive conditions: 90% were typical Y-shaped forks, 5% were ssDNA flaps with ssDNA tails with an average size of 300 nts, 3% resembled reversed forks, characterized by double-stranded regressed arms, and 3% were forks containing asymmetric gaps of less than 400 nts ssDNA at the fork branching point (Fig. 3a). Dna2-depleted cells exhibited a dramatic accumulation (40% versus 5%) of 10 times longer flapped molecules with an average size of 3000 nts ssDNA tails (Fig. 3a); we even found molecules with ssDNA flaps of 10.000 nts (Supplementary Fig. 2A). Concomitantly, in the absence of Dna2, Y-shaped molecules decreased to 40% while reversed and gapped forks increased to 10 and 5%, respectively (Fig. 3a). We note that the ssDNA flaps were rarely in the proximity of the fork branching point (Supplementary Fig. 2B), and, when that was the case, the ssDNA tails were shorter (Supplementary Fig. 2B). We conclude that the main phenotype of Dna2-depleted cells is the accumulation of aberrant and unprocessed flapped fragments behind the forks; moreover, these flaps persisted even when the bulk of DNA synthesis was completed, when cells reached G2. These long ssDNA tails can easily account for the DDR hyper-activation observed in the absence of Dna2 (Fig. 1a).

In a previous report, DNA flaps were visualized by TEM in *dna2*Δ freshly germinated spores from *S. pombe*^16^. While the frequency of DNA flaps (32%) in that study is in line with the present study, the average length of the ssDNA tails is much smaller (146 nt). This discrepancy can be ascribed to the different experimental conditions used in the two studies, such as the protocols used to enrich and stain the DNA fibers. Moreover, while in this study DNA flaps were detected in synchronous cells experiencing one single round of DNA replication without Dna2, in the previous study DNA flaps were visualized after the completion of the germination process in a non-defined stage of the cell cycle.

Slowing down fork speed, either by HU treatment or low temperature, restored normal Y-shaped forks in Dna2-depleted cells, nearly at the levels of control cells (Fig. 3b, c). Moreover, the frequencies of the flapped molecules reduced in Dna2-depleted cells, as a consequence of fork slow down, to 8 and 5% in HU and at low temperature, respectively, and the relative extent of the flapped ssDNA tails went from 3000 nts to less than 800 nts in both treatments (Fig. 3b, c). The *pif1-m2* mutation completely rescued the accumulation of aberrant RIs of Dna2-depleted cells (Fig. 3d). These data suggest that the high fork rate and Pif1 generate aberrant flapped intermediates in *dna2* mutants through a similar mechanism.

### Dna2 depletion does not cause fork pausing at natural pausing sites or chromosome fragmentation in the first cell cycle but continuous cell divisions in *dna2 rad9* cells lead to Pif1-dependent rDNA fragmentation

**Fig. 4:**
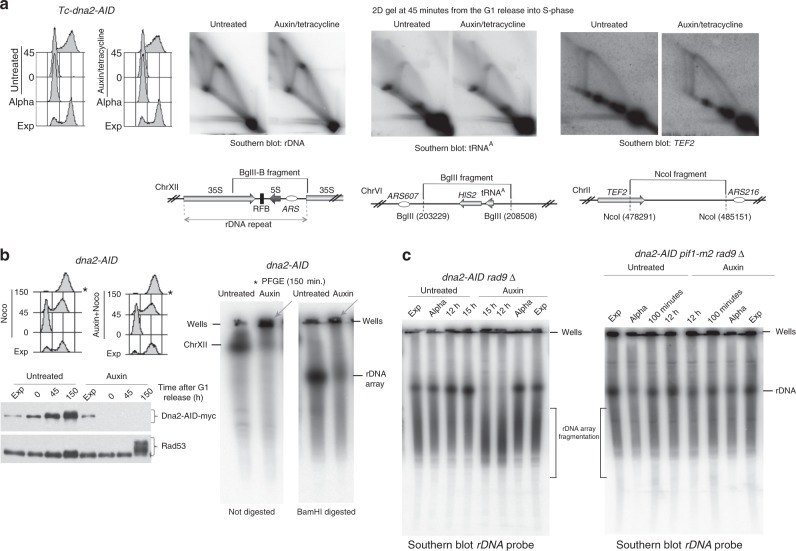
Dna2 depletion does not cause fork pausing in the first cell cycle but it induces Pif1-dependent rDNA fragmentation in *dna2-AID rad9* cells grown for 4-5 cell divisions in the absence of Dna2. **a** Neutral–neutral 2D gel electrophoresis of RIs at the three indicated natural pausing sites after a single unperturbed S phase in *Tc-dna2-AID* cells depleted (Auxin) or non-depleted (Untreated) for Dna2. **b** Chromosome XII and isolated rDNA array (*Bam*HI in plugs digestion^74^) migration patterns were analysed by pulsed field gel electrophoresis (PFGE) and Southern blotting in *dna2-AID* cells depleted (Auxin) or non-depleted (untreated) for Dna2 at 150 min from the G1 release into S phase. Cellular DNA content, dna2-AID-myc protein level, and Rad53 hyper-phosphorylation were detected, respectively, by FACS and western blotting. Grey arrows indicate retention of RIs into the wells of the PFGE gels. Position of the wells of the PFGE, Chromosome XII, and the rDNA array bands are indicated by black lines. **c** The length of the rDNA array was determined as in (**b**) at the indicated time points from the G1 release into S phase in *dna2-AID rad9*Δ and *dna2-AID pif1-m2 rad9*Δ cells depleted (Auxin) or non-depleted, (untreated) for Dna2. Fragmentation of the rDNA array is indicated by black brackets

We then analysed fork dynamics and chromosome fragility in *CL-dna2* cells depleted for Dna2 experiencing a single S phase and chromosome fragility in *dna2-AID rad9* cells after a defined number of cell divisions in the absence of Dna2. *CL-dna2* cells were released into S phase under depleting or non-depleting conditions and the RIs were analysed by neutral–neutral two-dimensional (2D) gel electrophoresis^48^ at three natural pausing sites, rDNA^49^, transfer RNA (tRNA^A^) ^50,51^, and *TEF2*^52^. We failed to detect significant differences at the level of 2D gel profiles in Dna2-depleted cells, compared to control cells (Fig. 4a). We note that a previous study reported accumulation of X-shaped molecules and DNA breakage in the rDNA of *dna2* hypo-morphic mutants grown for several cell divisions^53^, while in  this study we analyzed RIs and chromosome integrity in *CL-dna2* cells depleted for Dna2 experiencing one single round of DNA replication or chromosome fragmentation in  *CL-dna2 rad9* cells experiencing 4–5 cell divisions.

*FOB1* deletion did not suppress the cell lethality induced by Dna2 depletion (Supplementary Fig. 3), suggesting that fork pausing at the Fob1-dependent replication fork barrier at the rDNA *locus* is not the sole reason for *dna2* mutant’s sickness. rDNA fragmentation has been recently reported to be induced by mutations affecting replisome components^54^. We analysed, by PFGE, the fate of chromosome XII and the isolated rDNA array in Dna2-depleted cells experiencing one round of chromosome replication (Fig. 4b). Dna2-depleted cells accumulated branched chromosome XII and rDNA intermediates that were retained in the wells (Fig. 4b); we did not observe DNA fragmentation (Fig. 4b). Conversely, *CL-dna2 rad9* cells experiencing 5 rounds of DNA synthesis without Dna2 accumulated extensive rDNA fragmentation (Fig. 4c). Ablation of Pif1 (through the *pif1-m2* mutation) in *rad9*Δ *CL-dna2* cells depleted for Dna2 rescued rDNA fragmentation (Fig. 4c). Thus, Dna2 does not influence fork pausing but it is required to prevent the accumulation of branched RIs at rDNA during the first cell cycle and chromosome fragmentation when the cells replicate in the absence of Dna2 for several cell divisions. Rad9 acts as cell cycle barrier in counteracting the accumulation of DNA breaks in Dna2-depleted cells by blocking cell cycle progression.

## Discussion

**Fig. 5:**
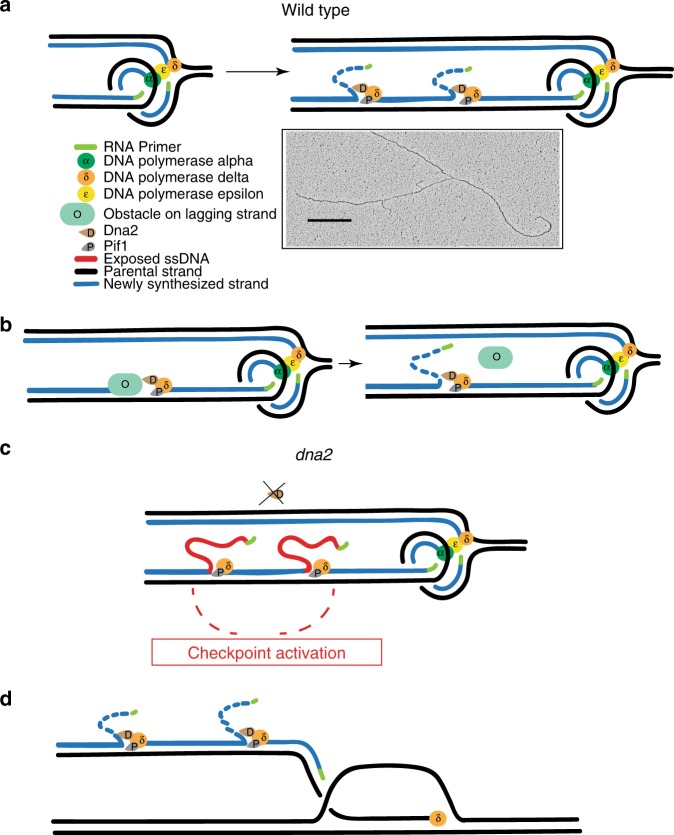
Model of the Polδ–Pif1–Dna2 assisted strand displacement and DNA flap cutting during DNA replication. **a** During unperturbed DNA replication extended and frequent Polδ–Pif1 strand displacement events create long DNA flaps, which are immediately cut by Dna2. The Polδ–Pif1–Dna2 machine might progress far from the fork branching point. EM picture of a rare fork with two DNA flaps in the proximity of the fork branching point. A black scale bar as in Fig. 3 is reported. **b** Re-priming coupled with Polδ–Pif1–Dna2 strand displacement may remove lagging strand DNA replication obstacles. **c** In the absence of Dna2,  Pif1-and fork speed-dependent DNA flaps accumulate on the lagging strand leading to checkpoint hyper-activation and cell death. If Dna2-depleted cells escape the first cell cycle arrest (*rad9*Δ condition) genome instability arises (rDNA fragmentation). **d** The Polδ–Pif1–Dna2 machine acts during conservative BIR-associated lagging strand DNA synthesis

Our data suggest that, in the absence of Dna2, the strand displacement mediated by Polδ and Pif1 frequently generates toxic long ssDNA flaps during lagging strand synthesis. Since Polα synthesizes short DNA segments, the displacement synthesis must go far beyond Polα products, particularly when flaps are not cleaved by Dna2. We propose that Dna2 plays a key role in chromosome replication to remove long ssDNA flaps generated by Polδ- and Pif1-dependent strand displacement of Okazaki fragments. Since Polα is not accurate, this mechanism may contribute to increase replication fidelity^55^. Extensive strand displacement can also be beneficial as it inversely correlates with the potential replication fork slippage of DNA polymerases^56^. In vitro studies simulating Okazaki fragments processing did not visualize long flaps in the absence of Pif1^2^, while when Pif1 was included in the reactions, ssDNA flaps ranged between 30 and 100 nt^23–25^. Interestingly, the Pif1–PCNA–Polδ complex can displace 2 kb DNA flaps in vitro^30^, and Polδ together with Pif1 can perform strand displacement for several kilobase pairs on gapped plasmids^21^. We propose that in a high fork speed mode Pif1–Polδ strand displacement is very efficient allowing DNA flaps to avoid Fen1 cutting. In this context Pif1 could easily unwind Okazaki fragments at the fork, possibly through its 5’–3’ directed ssDNA translocase activity^47^ and Dna2 would be essential to counteract the load of long DNA flaps. We note that our study does not contradict the idea that the majority of the Okazaki fragments are processed by the short flap pathway mediated by Fen1. Pif1- and Polδ-dependent strand displacement proceeds on the lagging strand with a direction opposite to the one of the moving fork; the “trombone” model has been proposed to coordinate leading and lagging strand synthesis with fork advance^57^. Our data suggest that the displacement events during lagging strand processing may proceed far from the fork branching point, thus implying that, occasionally, lagging strand processing becomes uncoupled from leading strand synthesis and fork advance. One possibility is that, while the leading and lagging polymerases proceed coupled at the fork branching point, occasionally, the lagging strand might experience additional polymerizing activities, behind the fork. This would imply that multiple DNA polymerases would act within the same template (Fig. 5a). Intriguingly, we have visualized intermediates containing more than one flap on the same filament of the fork (Fig. 5a). Taking into consideration that (i) strand displacement can proceed for long distances, at least in vitro^58^; (ii) T4 DNA polymerase and DNA helicase can carry on strand displacement with a speed of 400–500 base pairs per second with high processivity^59^; (iii) DNA replication fork speed in vivo was estimated to be three kilo bases *per minute* during an unperturbed S phase in *S. cerevisiae*^60^; and (iv) Polδ-dependent DNA synthesis has been shown to proceed for long distances during break-induced DNA replication (BIR)^61,62^, it is reasonable that the visualization of DNA flaps in the proximity of the fork branching points might be difficult under unperturbed conditions. The extended strand displacement activity may also remove bulky lagging strand obstacles that can be overcome by re-priming at the fork and removed, post-replicatively, by the backward directed Pif1–Polδ strand displacement activity^17,20^ (Fig. 5b). Accordingly, Pif1 removes Rap1 at telomeres, favouring Polδ-dependent strand displacement^21^. Another advantage of extensive strand displacement would be to peel off aberrant folded flaps that would be otherwise immune to cleavage by either Fen1 or Dna2^25^. It is also possible that discontinuous DNA synthesis on the lagging strand could create exceeding and toxic strand displacement events that must be immediately counteracted by Dna2 to prevent *futile* DNA damage checkpoint activation and unscheduled recombination events. While the Rad9 signalling is involved in monitoring the presence of ssDNA flaps generated in the absence of Dna2^40^, our data indicate that Rad9 ablation is not sufficient to rescue the lethality in cells deprived of Dna2, at least at high temperature and fork speed conditions. One possibility is that the lethal toxicity of the long ssDNA flaps generated in the absence of Dna2 is due to unscheduled genotoxic events, which lead to chromosome fragmentation (Fig. 5c). One round of S phase without Dna2 generates a small percentage of reversed forks. This could reflect either a role for Dna2 in counteracting or in restarting reversed forks^42,43^; however, our data suggest that, under unperturbed conditions, the major structural outcome of DNA replication without Dna2 is not fork reversal. Dna2 ablation also generates gapped forks, through a mechanism dependent on Pif1. Given that Pif1 has been shown to unwind tracts of lagging strand at the stalled fork when the checkpoint is defective^63^, and that Dna2 has been involved in the activation of the Mec1-mediated checkpoint^64^, one possibility is that, in Dna2-ablated cells, where the checkpoint is attenuated, the gapped forks are generated by sporadic and unscheduled Pif1-dependent unwinding activities at the lagging strand.

Dna2 also promotes DSB resection to assist homologous recombination^14,15^. The 3’ ssDNA resected tail can generate a D-loop structure, which initiates DNA replication through BIR^65^. BIR occurs through a conservative mechanism in which the D-loop is extended and migrates, the 3’end is elongated through leading strand synthesis, and the remaining ssDNA region outside the D-loop is replicated by the lagging strand apparatus^66,67^. This process implies uncoupling of leading and lagging strand synthesis. We speculate that the Dna2 replicative function has been hijacked to process the flaps generated during BIR-dependent strand displacement, mediated by Pif1 and Polδ. In this view, Dna2 does not promote a real resection process, rather it assists lagging strand synthesis during BIR (Fig. 5d).

## Methods

### Table of the strains

All the strains used in this study are listed in strains table 1 and are *W303* derivatives with the wild-type *RAD5* locus.

**Table 1:** Strains

Name	Genotype	Origin
TEMP17-I1	*MAT a, ade2-1, ura3-1, his3-11,15, leu2-3,112, trp1-1, can1-100, GAL, PSI* *+* *, leu2::GPD1-OsTIR1::LEU*	This study
TEMP17-F9	*MAT a, ade2-1, ura3-1, his3-11,15, leu2-3,112, trp1-1, can1-100, GAL, PSI* *+* , leu2::GPD1-OsTIR::LEU, dna2-AID*-9myc::Hyg	Branzei’s group
TEMP18-B4	*MAT a, ade2-1, ura3-1, his3-11,15, leu2-3,112, trp1-1, can1-100, GAL, PSI* *+* , leu2::GPD1-OsTIR::LEU, NAT-pADH1-tc3-dna2-AID*-9myc::hphNT	This study
TEMP17-G3	*MAT a, ade2-1, ura3-1, his3-11,15, leu2-3,112, trp1-1, can1-100, GAL, PSI* *+* , leu2::GPD1-OsTIR::LEU, dna2-AID*-9myc::Hyg, pif1-m2	This study
TEMP17-I5	*MAT a, ade2-1, ura3-1, his3-11,15, leu2-3,112, trp1-1, can1-100, GAL, PSI* *+* , leu2::GPD1-OsTIR::LEU, dna2-AID*-9myc::hphNT, rad9::KANMX6	This study
TEMP18-I2	*MAT a, ade2-1, ura3-1, his3-11,15, leu2-3,112, trp1-1, can1-100, GAL, PSI* *+* , leu2::GPD1-OsTIR::LEU, dna2-AID*-9myc::Hyg, fob1::HIS3	This study

### Culturing of *S. cerevisiae* cells and Dna2 degradation

*S. cerevisiae* cells were cultured at 28 °C or 20 °C in YP medium plus 2% of filtered glucose. G1 arrests were obtained by treating cell cultures with 4 µg/ml of alpha factor for 2 h. When indicated, the cells were released into the cell cycle in the presence of HU. The systems used in this study to generate conditional alleles of *DNA2* have been previously described^68–70^. Briefly, the tetracycline-dependent translational repressor (*Tc*) coding sequence was introduced in the non-coding N terminus of *DNA2*, while at its C terminus Dna2 was fused in frame with the auxin-dependent degron sequence AID^71–114^-9Myc. The host strain to induce auxin-dependent degradation of Dna2-AID expresses the Tir1 adaptor. Both *dna2-AID* and *Tc-dna2-AID* alleles induced rapid and complete degradation of the protein in 20 min after the addition of auxin and/or tetracycline. For the experiments presented in this study one or the other degron form of Dna2 have been used for convenience but the two alleles induce exactly the same phenotypes in all the tested conditions and degrade Dna2 with the same kinetic. Dna2 degradation was obtained by treating *Tc-dna2-AID* cells with 3-indole-acetic-acid sodium salt (IAA) (auxin-Abcam-ab146403) 1.95 × 10^-4^ g /ml and tetracycline 5.75 × 10^-4^ g/ml (Sigma T7660), while *dna2-AID* cells were treated only with auxin. In most of the experiments complete Dna2 degradation was achieved in 20 min during the last hour of alpha factor arrest when most of the cells were already in G1 to allow the study of the effects caused by a single S phase without Dna2.

### FACS analysis

Approximately 10^7^ cells were fixed with 100% ethanol for at least 1 h. Cells were centrifuged for 1 min at maximum speed, resuspended in 500 µl of Tris-HCL 50 mM pH 7.5 containing RNase A (1 mg/ml) (Sigma), and incubated overnight at 37 °C. Cells were treated with 500 µl of Tris-HCL 50 mM pH 7.5 containing Proteinase K (1 mg/ml) (Roche) for 1 h at 50 °C. Cells were then stained with propidium iodide (Sigma) 50 μg/ml in FACS Buffer solution (200 mM Tris-HCL pH 7.5, 200 mM NaCl, 80 mM MgCl_2_). A 1:10 dilution in Tris-HCL 50 mM pH 7.5 was sonicated and analysed in Becton Dickinson FACScalibur for FL2H fluorescence. For each sample, 10,000 events were counted and acquired data were analysed with CellQuest Software.

### SDS-PAGE electrophoresis

For protein extracts the cellular pellet of 20 ml of cell suspension (1 × 10^7^ cells/ml) was washed twice with 1 ml of 20% trichloroacetic acid (TCA) and suspended in 50 µl of 20% TCA. Cells were broken with acid-washed glass beads (Sigma G8772) on an ika vibrax vxr vortex for 5 min at maximum speed. After addition of 100 µl of TCA, 5% precipitated proteins were transferred into a new 1.5 ml tube and spun down in an Eppendorf centrifuge at 3000 rpm for 10 min at room temperature (RT). Excess of TCA was removed, and the pellets of proteins suspended in 100 µl of Laemmli buffer 1.5× (2× Laemmli Buffer is 4% SDS, 20% glycerol, 10% 2-mercaptoethanol, 0.004% bromophenol blue, and 0.125 M Tris-HCL pH 6.8). The pH was neutralized with 60 μl of Tris Base 2 M. The protein extract is boiled for 5 min at 95 °C and centrifuged for 2 min at top speed at RT. The supernatant is collected, and the protein extract was subjected to sodium dodecyl sulphate–polyacrylamide gel electrophoresis (SDS-PAGE) analysis. Discontinuous 10% SDS-PAGE gels were prepared with ratio acrylamide/bis-acrylamide (77:1) as described in table 2 and run in SDS-PAGE running buffer (Glycine 200 mM, Tris-HCL 25 mM, SDS 0.1%, pH 8.3) at constant current between 100 and 150 mA.

**Table 2:** SDS-PAGE gels

	Stacking gel	Running gel 10%
40% Acrylamide (Sigma 01697)	1.25 ml	5 ml
2% bis-acrylamide (Merck 106062)	0.7 ml	1.3 ml
0.5 M Tris-HCL pH = 6.8	2.5 ml	/
1.5 M Tris-HCL pH = 8.8	/	5 ml
SDS 10%	100 µl	200 µl
APS 10%	200 µl	200 µl
Temed 10%	20 µl	20 µl
Water until:	10 ml	20 ml

### Antibodies utilized for western blotting analysis

Antibody against Rad53 are mouse monoclonal, EL7 (IFOM) 50 µg/ml in serum-free medium (HB101, Irvine), used at a dilution of 1:200. Antibody against the myc epitope is mouse monoclonal 9E10 (IFOM) 1 mg/ml used at a dilution of 1:2000. Secondary antibodies are GAM (anti-mouse goat polyclonal) (Bio-Rad 170-6516) and IgG-HRP used at a dilution of 1:20,000. Antibodies against Β-subunit of DNA polymerase α primase are mouse monoclonal antibodies and are available upon request. Uncropped images of the films of the western blots presented in this study are reported in supplementary figures 4 and 5.

### Oligos and probes

Oligos to amplify the DNA cassettes necessary to introduce the *dna2-AID* and *Tc-dna2* conditional alleles by one-step replacement are reported: *dna2-AID* (gataaacctatcataaaggaaattctacaagagtatgaaagtcgtacgctgcaggtcgac and agctttcctgttatggagaagctcttcttattccccctgtcaatcgatgaattcgagctcg), *Tc-dna2* (ttatggcaaaacttgtgttacatttttgaagataaagttacagcataggccactagtggatctg and agatatactcgcagacctcttgttcttctgtggcgttccgggcatatgttctcgaggcctagg). DNA fragments used as probes for the southern blots presented in this study were amplified using the following couples of oligos: *rDNA* probe (gttgatcggacgggaaacggtg and gtgacaggtgccccgggtaaccc), *tRNA*^*A*^ probe (tcctcgaggtcatgcactcacaccattcacac and cagatgcggccgagtcggcgagcaaacaggg), *TEF2* probe (cagagatgatcgagccggtag and cctggcttgatgacaccgg).

### In vivo psoralen X-linking, purification of genomic DNA, and BND cellulose enrichment

The cells taken at the indicated time points and used to prepare the genomic DNA were fixed in 0.1% sodium azide in YP + 2% glucose for 30 min in ice. For the in vivo psoralen cross-linking cellular pellets kept in ice corresponding to 2–4 × 10^9^ cells were washed two times with cold sterile milliRX water and suspended in 5 ml of sterile cold water following a procedure previously described^71^. Briefly, four cycles of 10 min each of in vivo psoralen cross-linking were performed by placing the cell suspensions (transferred in 6-well plastic tissue-culture plates with flat bottoms) in a Stratagene Stratalinker 1800 irradiation chamber, under 365 nm (UV), with the plastic plates placed at 2–3 cm from the bulbs. Before each irradiation cycle, a fresh 0.3 ml aliquot of psoralen (4,5′,8 trimethyl-psoralen (TMP) 0.2 mg/ml dissolved in ethanol) was added to the cell suspension, which was mixed by pipetting and incubated for 5 min in the dark. Cell suspension was again mixed before each irradiation cycle. Plates containing the cell suspensions were placed on ice during the entire procedure. Cell suspensions were then transferred to 50 ml conical tubes, the pellets were washed twice with 30 ml of sterile cold water, and genomic DNA was extracted as follows. Spheroplasts are created by incubating the cellular pellet in 5 ml of spheroplasting buffer (Sorbitol 1 M, 100 mM EDTA, 50 mM Tris-HCL pH = 8, β-mercaptoethanol 14 mM and Zymolyase 100 T 1 mg/ml) for 30–60 min at 37 °C (pay attention do not overdigest the spheroplasts). Spheroplasts are centrifugated 3 min at 3000 rpm at 4 °C, the supernatant is carefully and completely eliminated and the spheroplasts pellet is resuspended with a 5 ml plastic pipette in 4 or 8 ml of P2 buffer (100 mM EDTA-NaOH pH = 8, 20 mM Tris-HCL pH = 7.6 and 1% SDS, 100 µl RNase A 10 mg/ml for 10 ml of buffer) and incubated at 50 °C for 10 min to allow complete dissolution of the spheroplasts. Then, 500 µl or 1 ml of Potassium Acetate 3 M pH = 5.5 are added to the lysis mix, which is mixed and incubated on ice for 30 min. The precipitation mix of each sample is divided and centrifugated in 2 ml Eppendorf tubes at 15k rpm for 30 min at 4 °C.

The supernatant is transferred into a 15 ml conical falcon tube and extracted with 3 or 6 ml of chloroform plus 0.5 ml of a mix of phenol/chloroform/isoamyl alcohol (24:24:1). The tubes are vortexed to allow extraction of the residual proteins. The tubes are centrifugated at room temperature at 4.5 K rpm and the upper aqueous phase is loaded directly onto a Qiagen genomic Tip100 G column previously equilibrated with QBT buffer (Qiagen). Two washes of the column are executed with 10 ml of QC (Qiagen) buffer and genomic DNA is eluted from the column with 5 ml of QF buffer into a 30 ml corex glass tube (Qiagen protocol). Then, 10 ml of isopropanol is added to each eluate, which is mixed by gently shaking the tube and incubating for 2 h at room temperature (or overnight). The precipitated DNA is centrifuged in a Beckmann centrifuge with the JS 13.1 swing rotor at 8500 rpm for 30 min at 10 °C. The pellet of genomic DNA is washed one time with 5 ml of Ethanol 70%. The residual ethanol is dried completely on the air, and the DNA pellet is resuspended in 250 µl of Tris-HCL 10 mM pH = 7.5–8. The genomic DNA can also be purified following the Qiagen protocol for the purification of yeast genomic DNA (Tip100 G columns). The purified yeast genomic DNA is partially digested with *Pvu*I (NEB) to reduce the size of the DNA fibers and to allow the EM analysis and ssDNA containing RIs are enriched on a column containing BND cellulose resin following the previously reported protocols^44^. The DNA flaps analysed in this study can be detected also on non BND enriched genomic DNA prepared with the Qiagen protocol for yeast genomic DNA (Tip100G columns).

### TEM analysis of RIs

We used in vivo psoralen X-linking coupled to BND cellulose enrichment, benzyldimethyl alkyl Ammonium Chloride (BAC) spreading of purified genomic DNA, DNA absorption on the carbon surface in the presence of ethidium bromide, uranyl acetate staining in the presence of ethanol, and low-angle platinum rotary shadowing to visualize DNA replication intermediates^44^. Briefly, a balanced mix of enriched genomic DNA cross-linked with psoralen was created with formamide and BAC. This mix was spread on a big surface of water with a specific spreading procedure. The mono-molecular film of DNA molecules on the surface of the water was touched with a 400-mesh copper TEM grid on which a thin (4–8 nm), homogeneous and low grain carbon layer was deposited. The carbon layer was created on a mica glass surface (2 cm × 2 cm) using the MED020 e-beam evaporator (Leica), equipped with the QSG monitor, two EK030 electron guns controlled by the EVM030 control unit. The e-beam evaporation parameters described in the instruction manual were used. The carbon layer deposited on the mica surface was floated on the surface of the water and transferred on the 400-mesh copper grids. The efficiency of the adsorption of the DNA molecules on the carbon surface was enhanced by a pre-treatment of the carbon surface deposited on the grid with a solution of ethidium bromide in water. Carbon grids with absorbed DNA molecules were immediately stained with a solution of uranyl acetate dissolved in ethanol and coated with 8 nm of Platinum using the MED020 evaporator modified with the low-angle grid shadowing kit (Leica 16770525) so that the sample holder was placed at an angle of 280.5 degrees and made an angle of around 3 degrees with the platinum gun fixed on the head of the instrument^44^. For platinum e-beam evaporation, we utilized the parameters indicated in the MED020 instruction manual. TEM pictures were taken using a FEI Tecnai12 Bio twin microscope operated at 80 KV and equipped with a side-mounted GATAN Orius SC-1000 camera controlled by the Digital Micrograph software. The files of the images in DM3 format were analysed using the ImageJ software. In these conditions the thickness of the DNA fibre is distributed around 10 nm^44^ and the conversion factor utilized to establish the length of the DNA molecules was 0.36 nm/base pair^71^.

### Neutral–neutral 2D gel analysis of RIs

Analysis of RIs through neutral–neutral 2D gel electrophoresis was carried on following previously reported protocols^71,72^. Briefly, 10–30 µg of DNA was digested with the specific restriction endonucleases, precipitated with potassium acetate and isopropanol, and resuspended in 10 mM Tris-HCL pH = 8. Digested genomic DNA was run on an OWL separation system model A2 electrophoresis chamber (gel tray 27 × 20 cm) filled with 2.5 litres of TBE (Tris-Borate-EDTA) buffer 1× not autoclaved. The first-dimension gel (500 ml; 0.35% w/v agarose) was prepared with TBE 1× not autoclaved and run at 50 volts for 24 h at room temperature. The second-dimension gel (500 ml;0.9% agarose w/v prepared with TBE 1× not autoclaved) was run in the same electrophoresis chamber with pre-chilled buffer at 150 volts for 12 h at 4 °C with current limited at 150 mA. Second-dimension gels were transferred on nylon filters through Southern blotting following standard procedures. Filters were subsequently hybridized with the indicated probes. Briefly, 50 ng of purified DNA probe was labelled with 50 µCi of α ^32^P dCTP using a random prime labelling kit (prime-a-gene labelling kit Promega) following the instruction manual. The labelling reaction was then passed through ProbeQuant^TM^ G-50 Micro Columns (GE Healthcare) following the manufacturer's instruction manual to remove the non-incorporated nucleotides. During the preparation of the radio-labelled probe, the membranes are rinsed with water and pre-hybridized with 20–30 ml of PerfectHyb^TM^ Plus Hybridization Solution (SIGMA) for at least 1 h at 65 °C in a rotating tube. The labelled probe was boiled 10 min at 95 °C and added to the pre-hybridization mix. The hybridization was performed at 65 °C overnight. The filters were washed for 30 min with 100 ml of 0.2× SSC, 0.1% SDS at 65 °C in a rotating tube and two times (30 min each) with 500 ml of 0.2× SSC, 0.1% SDS at 65 °C in a plastic tray with agitation. The hybridized membranes are briefly air-dried, covered with saran wrap, and exposed to a storage phosphor screen in an appropriate exposition cassette. The signals are analysed using Typhoon scanner (GE Healthcare) and quantified using ImageQuant program.

### PFGE to analyse yeast chromosomes and the rDNA array length

PFGE was performed as previously described^63,73^. Briefly agarose plugs have been run in a 1% agarose gel (Bio-Rad PFGE agarose) prepared in 150 ml of TBE 0.5× and the migration chamber was filled with 3 litres of TBE 0.5×. The Amersham Gene Navigator System was used with a water refrigerating bath set at 4 °C. The gel was run at 165 V for 24 h with 30 s pulses (step wise) for the separation of the chromosomes XII.

For the isolation of the rDNA array genomic DNA embedded in the plugs was digested with *Bam*HI^74^ and digested plugs were run into the PFGE as described above using the following run conditions :100 V for 69 h (step 1 = 68 h with pulses of 300 s and step 2 = 1 h with 900 s pulses; the two steps were connected by the interpolation mode). During the PFGE run pulses went from 300 to 900 s.

### Reporting summary

Further information on experimental design is available in the Nature Research Reporting Summary linked to this paper.

## Electronic supplementary material

Supplementary Information

Peer Review File

Reporting Summary

## Data Availability

The data generated and/or analysed during the current study are available from the corresponding authors on reasonable request.
