# Supplementary material for: Dna2 processes behind the fork long ssDNA flaps generated by Pif1 and replication-dependent strand displacement
Source: Nat Commun. 2018 Nov 16;9:4830. doi: 10.1038/s41467-018-07378-5 (PMC6240037; doi:10.1038/s41467-018-07378-5)
Supplement: Supplementary file 1 — Supplementary Information [file 41467_2018_7378_MOESM1_ESM.pdf]

Supplementary information.

"Dna2 processes behind the fork long ssDNA flaps generated by Pif1 and replication-dependent strand displacement." Rossi et al 2018.

Supplementary figure 1

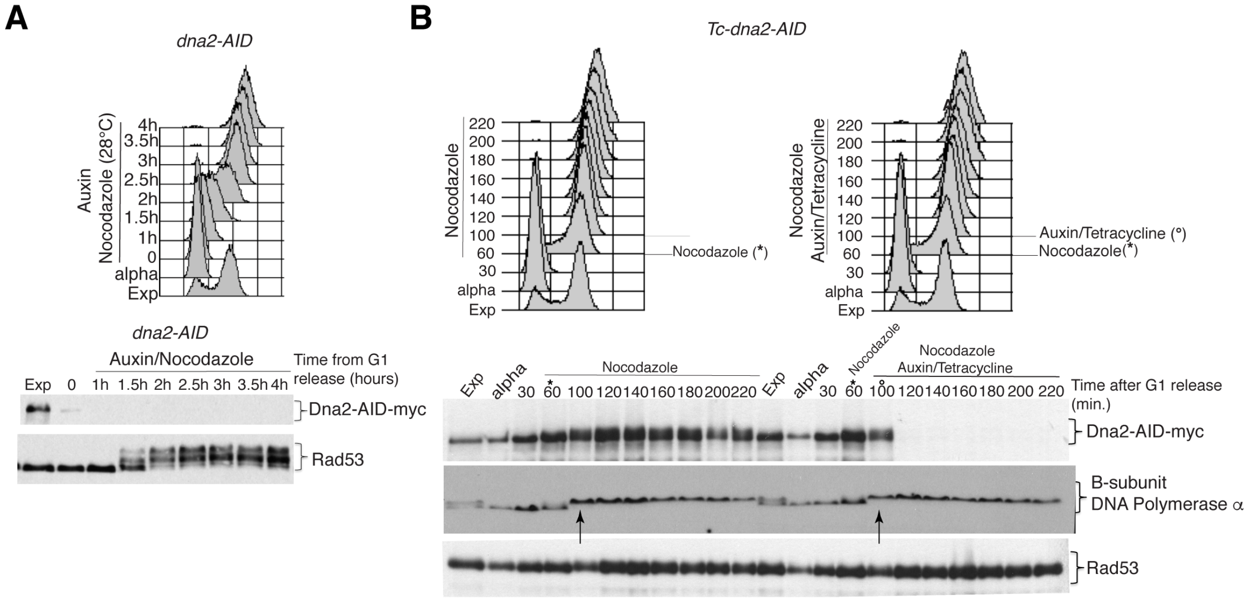

Supplementary Figure 1 (Refers to Figure 1). DDR hyper-activation in the absence of Dna2 depends upon S-phase. A) Dna2 was depleted in G1 arrested *dna2-AID* cells. Cells were released into S-phase in the presence of nocodazole and auxin. Cellular DNA content, *dna2-AID-myc* protein levels and Rad53 phosphorylation were detected by FACS and western blotting. B) *Tc-dna2-AID* cells arrested in G1 were released in S-phase in the presence of nocodazole. After 100 minutes from the G1 release (when cells already reached the 2C DNA content), auxin and tetracycline were added to induce degradation of Dna2 after S-phase completion. Cells in which auxin and tetracycline were not added were kept in parallel as control. Rad53 and Pol α B-subunit were monitored by western blotting.

Supplementary figure 2

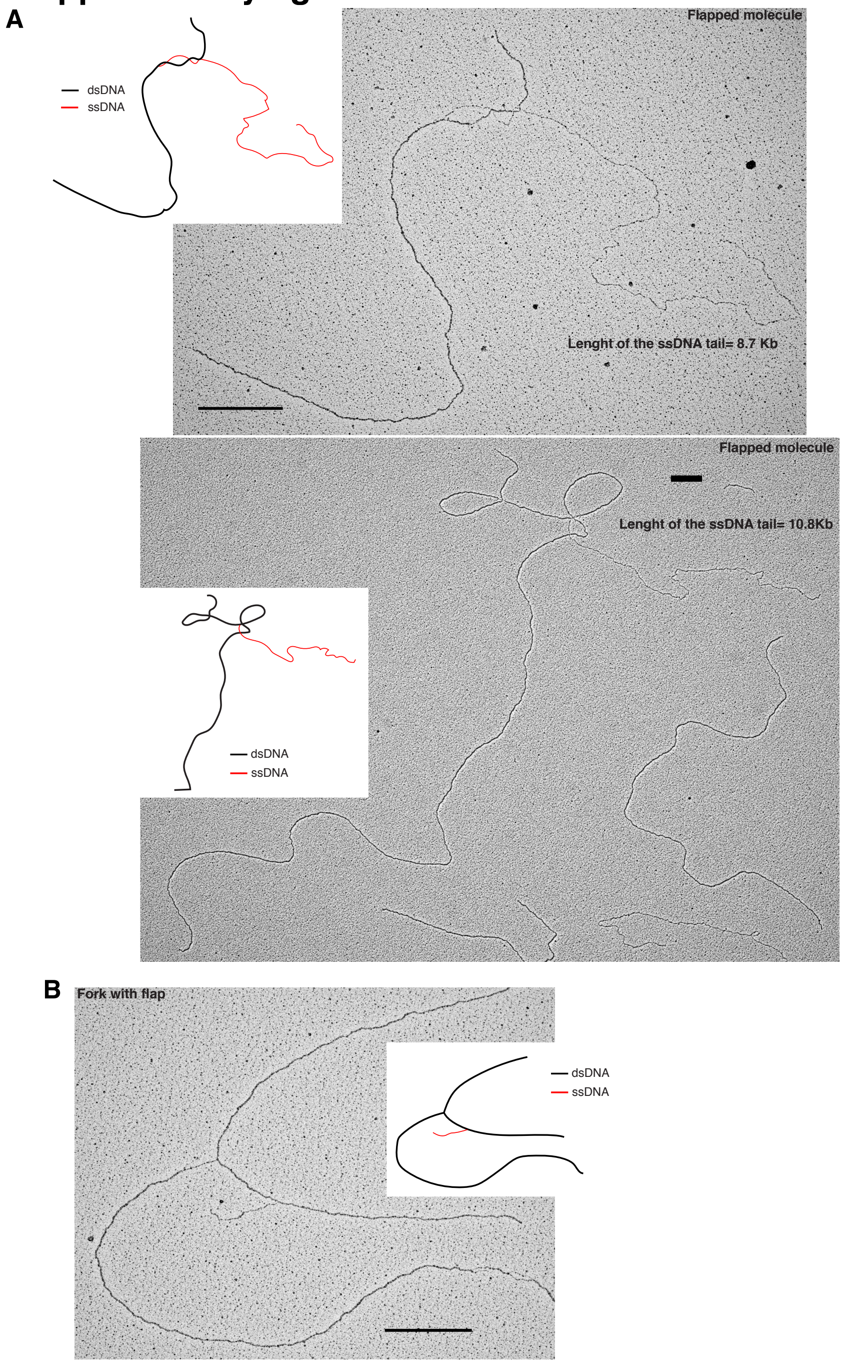

Supplementary Figure 2 (Refers to Figure 3). DNA flaps with 8-10Kb ssDNA tails accumulate following a single unperturbed S-phase without Dna2. A) Two representative EM pictures of flapped molecules with DNA flaps with ssDNA tails with lengths between 8 and 10 kilobases. Molecules were isolated and analysed in the experiments described in Figure 3A-B. B) Representative EM picture of a rare RI with a DNA flap in close proximity of the DNA replication fork branching point. A schematic representation of each DNA structure is reported with dsDNA in black and ssDNA in red. Black scales bars of 360nm, which correspond to 1 kilobase of DNA are reported on each picture.

Supplementary figure 3

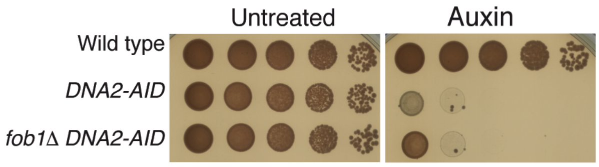

Supplementary Figure 3 (Refers to Figure 4). Cell lethality induced by Dna2 ablation does not depend upon Fob1 dependent replication fork barrier. Cell survival in the absence and presence of Auxin has been analysed for the indicated yeast strains by drop assay.

Supplementary figure 4

Refer to figure 1A

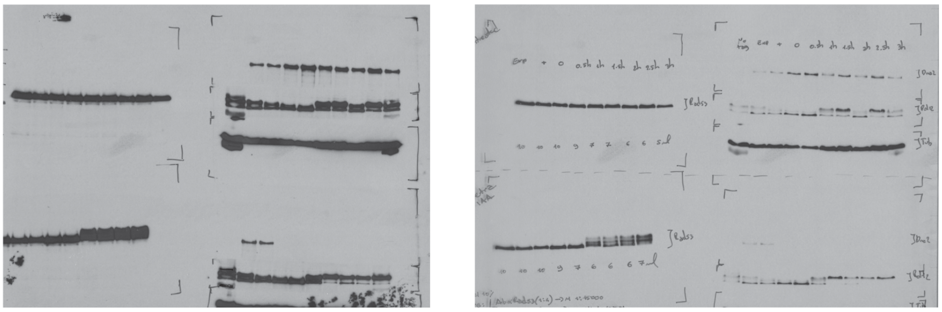

Refer to figure 1B

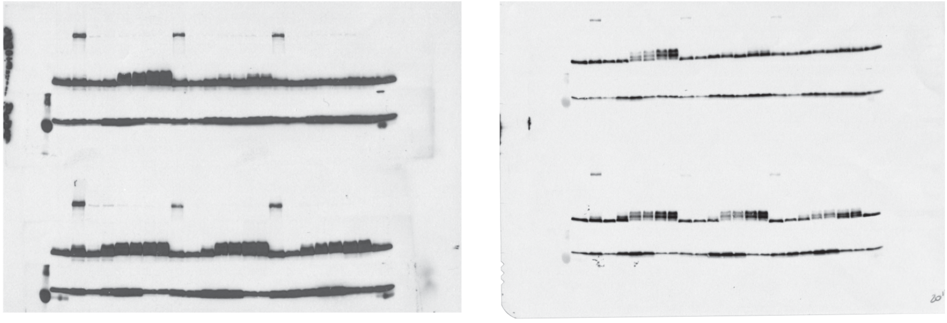

Refer to figure 1C

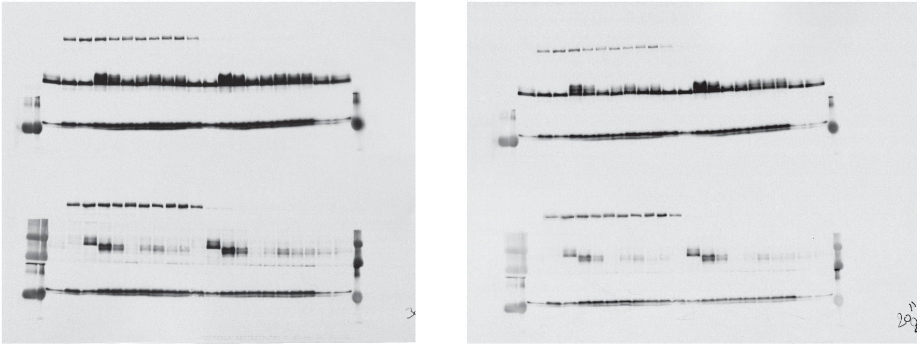

Refer to figure 1D

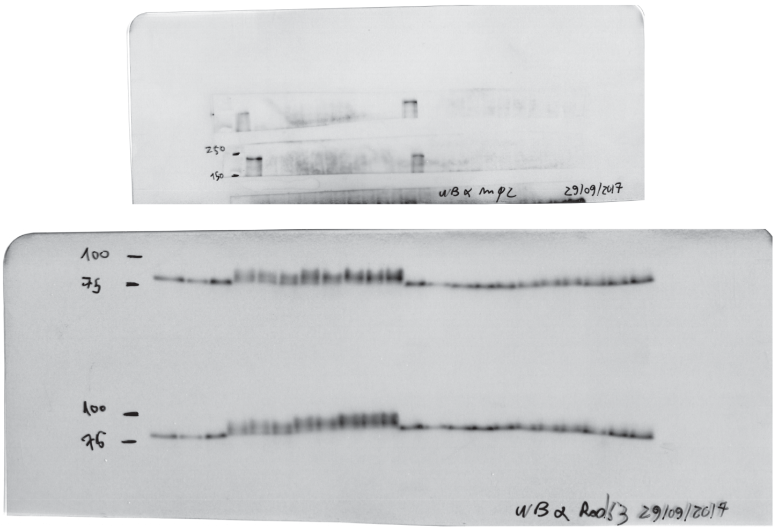

Supplementary Figure 4. Raw images of the western blots used in Figure 1.

Supplementary figure 5

Refer to figure 4B

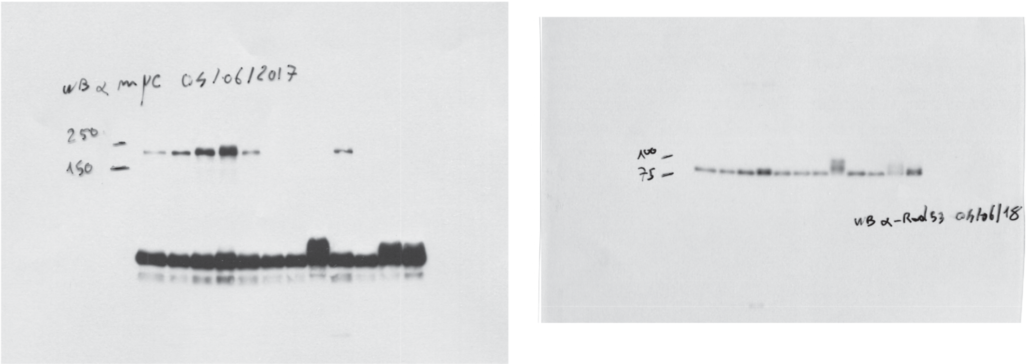

Refer to supplementary figure 1A

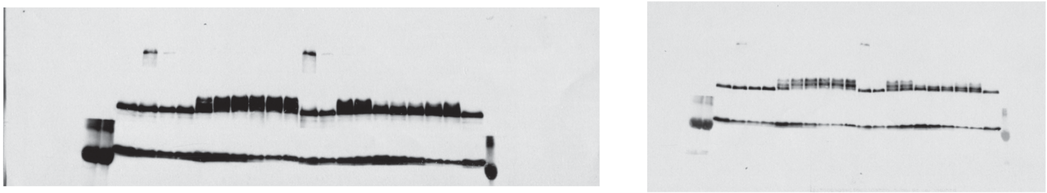

Refer to supplementary figure 1B

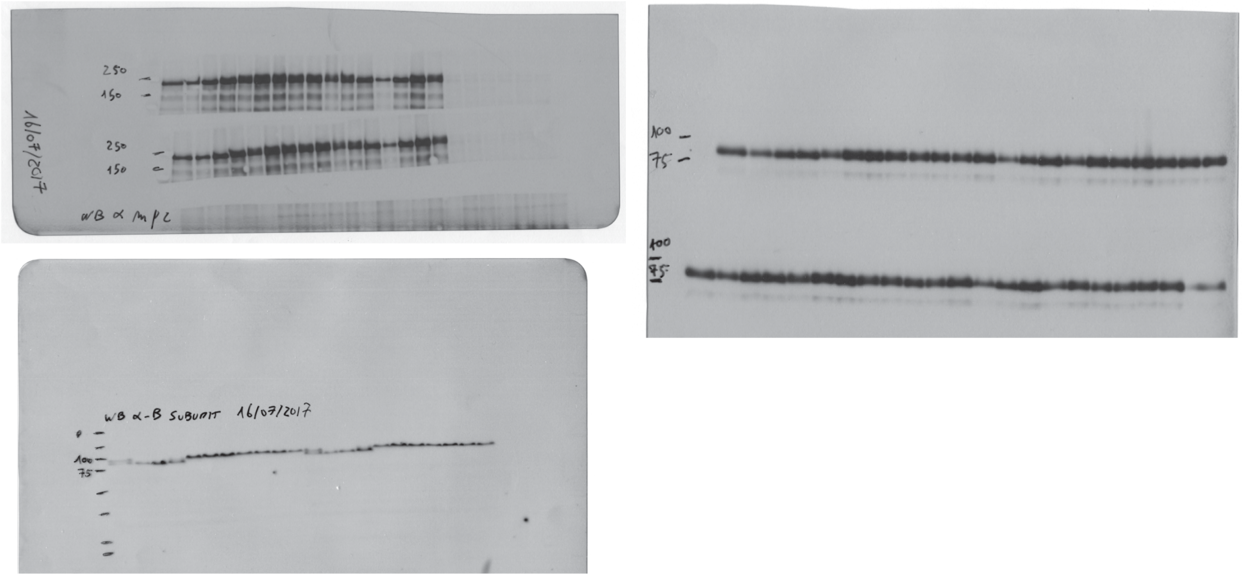

Supplementary Figure 5. Raw images of the western blots used in Figure 4 and supplementary Figure 1.
