# Supplementary material for: Dna2 processes behind the fork long ssDNA flaps generated by Pif1 and replication-dependent strand displacement
Source: Nat Commun. 2018 Nov 16;9:4830. doi: 10.1038/s41467-018-07378-5 (PMC6240037; doi:10.1038/s41467-018-07378-5)
Supplement: Supplementary file 3 — Reporting Summary [file 41467_2018_7378_MOESM3_ESM.pdf]

nature research

Corresponding author(s): NCOMMS-18-25835-T Michele Giannattasio
Marco Foiani

# Reporting Summary

Nature Research wishes to improve the reproducibility of the work that we publish. This form provides structure for consistency and transparency in reporting. For further information on Nature Research policies, see Authors & Referees and the Editorial Policy Checklist.

## Statistical parameters

When statistical analyses are reported, confirm that the following items are present in the relevant location (e.g. figure legend, table legend, main text, or Methods section).

| n/a | Confirmed | |
|---|---|---|
| ☐ | ☒ | The exact sample size (*n*) for each experimental group/condition, given as a discrete number and unit of measurement |
| ☐ | ☒ | An indication of whether measurements were taken from distinct samples or whether the same sample was measured repeatedly |
| ☒ | ☐ | The statistical test(s) used AND whether they are one- or two-sided<br>*Only common tests should be described solely by name; describe more complex techniques in the Methods section.* |
| ☒ | ☐ | A description of all covariates tested |
| ☒ | ☐ | A description of any assumptions or corrections, such as tests of normality and adjustment for multiple comparisons |
| ☐ | ☒ | A full description of the statistics including central tendency (e.g. means) or other basic estimates (e.g. regression coefficient) AND variation (e.g. standard deviation) or associated estimates of uncertainty (e.g. confidence intervals) |
| ☒ | ☐ | For null hypothesis testing, the test statistic (e.g. *F*, *t*, *r*) with confidence intervals, effect sizes, degrees of freedom and *P* value noted<br>*Give P values as exact values whenever suitable.* |
| ☒ | ☐ | For Bayesian analysis, information on the choice of priors and Markov chain Monte Carlo settings |
| ☒ | ☐ | For hierarchical and complex designs, identification of the appropriate level for tests and full reporting of outcomes |
| ☒ | ☐ | Estimates of effect sizes (e.g. Cohen's *d*, Pearson's *r*), indicating how they were calculated |
| ☐ | ☒ | Clearly defined error bars<br>*State explicitly what error bars represent (e.g. SD, SE, CI)* |

*Our web collection on statistics for biologists may be useful.*

## Software and code

Policy information about availability of computer code

| | |
|---|---|
| Data collection | Digital Micrograph (Gatan) was used for the acquisition of the TEM pictures |
| Data analysis | ImageJ-NIH was used to meausure the DNA molecules |

For manuscripts utilizing custom algorithms or software that are central to the research but not yet described in published literature, software must be made available to editors/reviewers upon request. We strongly encourage code deposition in a community repository (e.g. GitHub). See the Nature Research guidelines for submitting code & software for further information.

## Data

Policy information about availability of data

All manuscripts must include a data availability statement. This statement should provide the following information, where applicable:

- Accession codes, unique identifiers, or web links for publicly available datasets
- A list of figures that have associated raw data
- A description of any restrictions on data availability

There is no restriction on data availability.

# Field-specific reporting

Please select the best fit for your research. If you are not sure, read the appropriate sections before making your selection.

☒ Life sciences ☐ Behavioural & social sciences ☐ Ecological, evolutionary & environmental sciences

For a reference copy of the document with all sections, see nature.com/authors/policies/ReportingSummary-flat.pdf

# Life sciences study design

All studies must disclose on these points even when the disclosure is negative.

| | |
|---|---|
| Sample size | Sample size for the electron microscopy was chosen based on previously reported protocols: Zellweger, R. & Lopes, M. Dynamic Architecture of Eukaryotic DNA Replication Forks In Vivo, Visualized by Electron Microscopy.<br>Methods Mol Biol 1672, 261-294, doi:10.1007/978-1-4939-7306-4_19 (2018). |
| Data exclusions | no data were excluded |
| Replication | all attempts at replication were successful |
| Randomization | not applicable |
| Blinding | not applicable |

# Reporting for specific materials, systems and methods

## Materials & experimental systems

| n/a | Involved in the study | |
|---|---|---|
| ☒ | ☐ | Unique biological materials |
| ☐ | ☒ | Antibodies |
| ☒ | ☐ | Eukaryotic cell lines |
| ☒ | ☐ | Palaeontology |
| ☒ | ☐ | Animals and other organisms |
| ☒ | ☐ | Human research participants |

## Methods

| n/a | Involved in the study | |
|---|---|---|
| ☒ | ☐ | ChIP-seq |
| ☐ | ☒ | Flow cytometry |
| ☒ | ☐ | MRI-based neuroimaging |

## Antibodies

| | |
|---|---|
| Antibodies used | Antibody against Rad53 are mouse monoclonal, EL7 (IFOM): Fiorani S, Mimun G, Caleca L, Piccini D, Pellicioli A.<br>Cell Cycle. 2008 Feb 15;7(4):493-9.Characterization of the activation domain of the Rad53 checkpoint kinase.<br>Anti myc epitope antibodies are mouse monoclonal (9E10) (Santa Cruz Botechnology #Sc-40 ). |
| Validation | Antibodies used in this study have been validated in the above mentioned publications or manufacturer data sheets. |

## Flow Cytometry

### Plots

Confirm that:

☒ The axis labels state the marker and fluorochrome used (e.g. CD4-FITC).

☒ The axis scales are clearly visible. Include numbers along axes only for bottom left plot of group (a 'group' is an analysis of identical markers).

☒ All plots are contour plots with outliers or pseudocolor plots.

☒ A numerical value for number of cells or percentage (with statistics) is provided.

### Methodology

| | |
|---|---|
| Sample preparation | sample preparation has been described in materials and methods section |

| | |
|---|---|
| Instrument | Becton Dickinson FACSCalibur |
| Software | Becton Dickinson CellQuest |
| Cell population abundance | Homogeneous Yeast cell populations were used. Checked at the optical microscope. |
| Gating strategy | no gating strategy was applied |

☐ Tick this box to confirm that a figure exemplifying the gating strategy is provided in the Supplementary Information.
