# Supplementary material for: Dna2 processes behind the fork long ssDNA flaps generated by Pif1 and replication-dependent strand displacement
Source: Nat Commun. 2018 Nov 16;9:4830. doi: 10.1038/s41467-018-07378-5 (PMC6240037; doi:10.1038/s41467-018-07378-5)
Supplement: Supplementary file 2 — Peer Review File [file 41467_2018_7378_MOESM2_ESM.pdf]

Editorial Note: This manuscript has been previously reviewed at another journal that is not operating a transparent peer review scheme. The manuscript was considered suitable for publication without further review at Nature Communications.
